# Allied health professionals’ experiences of co-worker unprofessional behaviour and their reported speaking-up skills: A secondary analysis of a cross-sectional survey

**DOI:** 10.1016/j.fhj.2025.100262

**Published:** 2025-06-06

**Authors:** Ryan D. McMullan, Tim Badgery-Parker, Ling Li, Rachel Urwin, Johanna I. Westbrook

**Affiliations:** Australian Institute of Health Innovation, Faculty of Medicine, Health and Human Sciences, Macquarie University, Level 6, 75 Talavera Road, New South Wales, Australia

**Keywords:** Allied health, Bullying, Incivility, Speaking-up, Unprofessional behaviour

## Abstract

Unprofessional behaviour among hospital staff is prevalent worldwide. Few studies have investigated the experiences of allied health hospital staff. The objective of our study was to examine the types and prevalence of unprofessional behaviour experienced by these staff as well as their self-reported speaking-up skills. We performed a secondary analysis of survey data from staff across seven hospitals. Approximately 92% (*N*=485; 95% CI, 89.6–93.9) of respondents experienced incivility or bullying at least once in the preceding 12 months, and 29.7% (*N*=157; 95% CI, 25.5–34.0) experienced incivility or bullying weekly to multiple times daily. Respondents who reported having speaking-up skills experienced less frequent incivility or bullying (OR, 0.53; 95% CI, 0.35–0.81) than those staff without these skills. Speaking-up programmes may better support staff to address unprofessional behaviour if implemented as part of hospital-wide culture change interventions involving all hospital staff.

## Highlights

• Approximately 92% of allied health professionals experienced incivility or bullying.
• Approximately 30% experienced incivility or bullying weekly to multiple times daily.
• Respondents who reported having speaking-up skills experienced less frequent incivility or bullying.
• Speaking-up programmes may better support staff to address unprofessional behaviour.

## Introduction

Allied health professionals provide essential care to patients in hospitals and are core members of the clinical team. Allied health professionals comprise a range of professionals, including physiotherapists, pharmacists, speech pathologists, psychologists and occupational therapists. They make up a large part of the healthcare workforce in Australia. In 2022, there were a reported 74,601 diagnostic and allied health professionals in public hospitals, making up 16.6% of total hospital staff.^1^ While many studies have investigated the experiences of medical and nursing staff about unprofessional behaviour such as bullying and harassment,^2^, ^3^, ^4^, ^5^ very few studies have investigated the experiences of allied health professionals. A small study in Australia over 10 years ago found that 24% of 166 allied health respondents had experienced workplace bullying.^6^ These experiences were associated with higher levels of depression and psychological distress.^6^

Unprofessional behaviour can range from incivility or bullying to more overtly hostile behaviour such as physical and sexual assault. These behaviours can have a negative impact on staff wellbeing with studies finding associations with depression, suicidal ideation, reduced morale, increased sick leave and burnout.^7^, ^8^, ^9^ In 2019, bullying and harassment was estimated to cost the NHS in the UK £2.281 billion per annum due to sickness absence, staff turnover, diminished productivity and litigation.^10^

Unprofessional behaviour also threatens patient safety.^11^, ^12^, ^13^, ^14^, ^15^ A US study of 202 surgeons and 13,653 patients found that patient medical and surgical complication risk was higher for those whose surgeons had a higher number of co-worker reports about unprofessional behaviour compared with patients whose surgeons had fewer reports.^13^ Staff reports of unprofessional behaviours by colleagues, including ‘opinions being ignored’ and ‘someone withholding information which affects work performance’, have been associated with increased risks to patient safety in a sample of eight Australian hospitals.^14^

Given the diverse impact of unprofessional behaviour on staff wellbeing, organisational costs, and patient safety, health systems in the USA, UK and Australia have invested in culture change interventions which include programmes to encourage speaking up.^16^, ^17^, ^18^, ^19^ Speaking up refers to effectively communicating concerns, ideas or questions in response to issues or behaviours that threaten teamwork and the safe delivery of care to patients. Freedom To Speak Up Guardians (FTSUG) were established in the NHS with the overall goal of supporting staff raise concerns about problems in healthcare services.^19^ An evaluation of FTSUG revealed that 45% of 7,000 instances of speaking up were related to unprofessional behaviour.^19^ This unexpected finding further demonstrates the inter-connection between unprofessional behaviour and patient safety and reinforces the need for resources and processes for staff to support them to address unprofessional behaviour. However, evidence of the effectiveness of speaking-up programmes is inconclusive.^20^ The extent to which speaking up reduces the rate of unprofessional behaviour experienced by staff is not clear.

Allied health professionals’ experiences of unprofessional behaviour as well as their experiences of speaking up have received little attention. Knowledge of their experiences can better inform organisational culture change programmes to address unprofessional behaviour hospital-wide, beyond the predominant focus on the behaviours of doctors and nurses. Allied health professionals are critical members of clinical teams with essential roles in the continuum of care involved in assessing, diagnosing, treating and discharging patients. Their experiences of unprofessional behaviour may equally negatively affect clinical teamwork and jeopardise patient care.

We aimed to (i) quantify the types and prevalence of unprofessional behaviour experienced by allied health professionals, (ii) identify respondent characteristics associated with the experience of unprofessional behaviour, and (iii) examine reported speaking-up skills and associations between speaking-up skills and experience of unprofessional behaviour.

## Methods

### Study design

A cross-sectional survey was conducted in 2017/2018 as part of a larger programme of research evaluating a hospital-wide professional accountability programme.^2^^,^^14^^,^^17^^,^^21^, ^22^, ^23^, ^24^ The secondary analysis reported here includes data from this survey.^2^ The project was approved by the Human Research Ethics Committee of St Vincent’s Hospital Melbourne (reference: HREC/17/SVHM/237).

### LION survey

The Longitudinal Investigation Of Negative behaviour (LION) survey examines 26 unprofessional behaviours from incivility (eg being spoken to rudely) to physical and sexual assault experienced in the previous 12 months (Appendix A).^2^ The survey incorporates questions from the Negative Acts Questionnaire Revised and the Royal Australasian College of Surgeons Discrimination, Bullying and Sexual Harassment Survey.^25^^,^^26^ The survey was piloted and tested for face validity with a convenience sample of health professionals.^2^ The survey asked participants to indicate the frequency of experiencing the 26 unprofessional behaviours during the preceding 12 months (seven-point scale, with endpoints labelled ‘never’ to ‘multiple times a day’). The survey also included questions about speaking-up skills and respondent age, gender, length of employment at the hospital and in the health sector, and professional group. Speaking-up questions ask respondents the extent to which they agree or disagree (five-point scale: strongly disagree, disagree, neither agree nor disagree, agree, strongly agree) with 11 statements about their skills to effectively speak up about unprofessional behaviour, whether they are encouraged by their colleagues to speak up, and whether they know the proper channels to raise a concern. Allied health professionals comprised pharmacy; physiotherapy; occupational therapy; dietician; and social, welfare or pastoral care workers.

### Participants

The LION Survey was distributed to all hospital staff in seven Australian hospitals (both public and private hospitals) across three states with details previously reported.^2^ The analysis reported here is a secondary analysis of responses to the survey from allied health professionals.

### Data analysis

For the descriptive analysis, 21 of the LION Survey items were categorised as incivility/bullying. For this category we categorised weekly, daily, or several times daily as ‘frequent’; once or twice a year, every few months, or about monthly as ‘occasional’, and ‘never’. The five ‘extreme’ unprofessional behaviours (threats of violence; inappropriate or unwanted touching; demands for sexual favours; sexual assault; physical assault) were classified as ‘ever’ or ‘never’. Missing and ‘prefer not to answer’ responses were grouped together as ‘missing’. These categories are consistent with those reported in our previous studies.^2^ For the descriptive analysis, we calculated the percentage of respondents in each response category overall and by relevant subgroups. We calculated simultaneous multinomial 95% confidence intervals for the percentages.^27^

We used multivariable regression models to examine the associations between respondent characteristics and three main outcomes of interest: (i) experience of unprofessional behaviour (incivility or bullying) (frequent versus never/occasional) – a binary logistic model, (ii) experience of extreme unprofessional behaviour (ever versus never) – a binary logistic model, and (iii) speaking up (being comfortable with speaking up and having the skills to speak up if experiencing unprofessional behaviour) (strongly agree or agree versus neither disagree or agree, disagree or strongly disagree) – a binary logistic model. Each model used available complete cases (ie surveys with missing data for any variable in the model were excluded). As some subcategories in our data had 0 responses and the number of extreme unprofessional behaviours was small, we used Firth penalised logistic regression for analysis.^28^ This method applies a bias-reduction technique to the maximum likelihood estimates, making it particularly suitable for rare event data and small samples. The Firth correction prevents infinite parameter estimates in the presence of complete or quasi-complete separation while also reducing small-sample bias in the estimated odds ratios. Models included participant age, gender, role and hospital. Results are presented as odds ratios with 95% confidence intervals (CIs). We used SAS 9.4 for data cleaning and R 4.3 for analysis.

## Results

### Respondent characteristics

The response rate was 37.3% (529 out of 1,418) among all allied health professionals across study hospitals. Characteristics of respondents are shown in Table 1.

**Table 1:** Respondent characteristics for allied health professionals (*N*=529).

Characteristic	Allied health (*N*, %)
Age (years)	
18–24	22 (4.2%)
25–34	205 (38.8%)
35–44	137 (25.9%)
45–54	82 (15.5%)
≥55	79 (14.9%)
Prefer not to answer	4 (0.8%)
Gender	
Male	85 (16.1%)
Female	437 (82.6%)
Other	1 (0.2%)
Prefer not to answer	6 (1.1%)
Hospital	
A	228 (43.1%)
B	45 (8.5%)
C	196 (37.1%)
D	12 (2.3%)
E	15 (2.8%)
F	14 (2.6%)
G	19 (3.6%)
Time employed in hospital	
<1 yr	74 (14.0%)
1–2 yrs	95 (18.0%)
3–5 yrs	155 (29.3%)
6–10 yrs	103 (19.5%)
11–20 yrs	68 (12.9%)
20+ yrs	30 (5.7%)
Missing	4 (0.8%)
Time employed in sector	
<1 yr	18 (3.4%)
1–2 yrs	28 (5.3%)
3–5 yrs	86 (16.3%)
6–10 yrs	138 (26.1%)
11–20 yrs	136 (25.7%)
20+ yrs	120 (22.7%)
Missing	3 (0.6%)

### Prevalence of unprofessional behaviour

Of the 529 allied health respondents, 91.7% (*N*=485; 95% CI, 89.6–93.9) experienced incivility or bullying at least once in the preceding 12 months and 29.7% (*N*=157; 95% CI, 25.5–34.0) experienced incivility or bullying weekly to multiple times daily. The most frequently experienced unprofessional behaviour was ‘opinions being ignored’, with 11.7% (*N*=62; 95% CI, 7.8–15.7) of staff experiencing this frequently and 67.1% (*N*=355; 95% CI, 63.1–71.1) experiencing it occasionally (Table 2). Approximately 7% (*N*=38; 95% CI, 5.1–9.3) of respondents reported experiencing one or more extreme unprofessional behaviours in the preceding 12 months, of which 57.9% (*N*=22) experienced ‘Inappropriate or unwanted touching’.

**Table 2:** Experience of unprofessional behaviour by allied health professionals (*N*=529).

	Experience of these behaviours in the last 12 months			
Incivility or bullying behaviours	Frequent	Occasional	Never	Missing/Prefer not to answer
	Weekly, daily, several times daily	Once or twice a year, every few months, about monthly		
	*N* (% of respondents)	*N* (% of respondents)	*N* (% of respondents)	*N* (% of respondents)
Being given unreasonable workload/deadlines	65 (12.3%)	233 (44%)	226 (42.7%)	5 (0.9%)
Opinions being ignored	62 (11.7%)	355 (67.1%)	108 (20.4%)	4 (0.8%)
Being spoken to rudely	59 (11.2%)	352 (66.5%)	117 (22.1%)	1 (0.2%)
Excessive monitoring of work	47 (8.9%)	133 (25.1%)	347 (65.6%)	2 (0.4%)
Being ignored or excluded	32 (6.0%)	182 (34.4%)	312 (59.0%)	3 (0.6%)
Someone withholding information which affects work performance	30 (5.7%)	224 (42.3%)	270 (51.0%)	5 (0.9%)
Repeated reminders of errors or mistakes	24 (4.5%)	150 (28.4%)	351 (66.4%)	4 (0.8%)
Being shouted at or being the target of anger	16 (3.0%)	174 (32.9%)	336 (63.5%)	3 (0.6%)
Being the subject of excessive teasing/sarcasm	11 (2.1%)	64 (12.1%)	449 (84.9%)	5 (0.9%)
Physically intimidating behaviours	10 (1.9%)	95 (18.0%)	421 (79.6%)	3 (0.6%)
Hints or signals from others to quit your job	10 (1.9%)	57 (10.8%)	458 (86.6%)	4 (0.8%)
Being told sexually explicit or offensive jokes/comments at work	8 (1.5%)	100 (18.9%)	420 (79.4%)	1 (0.2%)
Being humiliated or ridiculed	8 (1.5%)	81 (15.3%)	438 (82.8%)	2 (0.4%)
Having key areas of responsibility removed or replaced with meaningless or unpleasant tasks	8 (1.5%)	70 (13.2%)	448 (84.7%)	3 (0.6%)
Treated unfairly based on gender, ethnicity, sexual orientation, religion, disability, pregnancy, parenting responsibilities, age	7 (1.3%)	52 (9.8%)	466 (88.1%)	4 (0.8%)
Negative comments or offensive jokes about gender, ethnicity, sexual orientation, religion, disability, pregnancy, parenting responsibilities, age	4 (0.8%)	70 (13.2%)	452 (85.4%)	3 (0.6%)
Unwelcome practical jokes	4 (0.8%)	37 (7.0%)	485 (91.7%)	3 (0.6%)
Graphic comments/questions/ insinuations about appearance, sexual or private life	3 (0.6%)	69 (13%)	456 (86.2%)	1 (0.2%)
Having unjustified allegations made	2 (0.4%)	74 (14.0%)	449 (84.9%)	4 (0.8%)
Unwelcome sexual flirtations/persistent requests for dates	0 (0.0%)	11 (2.1%)	518 (97.9%)	0 (0.0%)
Being shown sexually suggestive photos, videos, emails or texts	0 (0.0%)	9 (1.7%)	520 (98.3%)	0 (0.0%)
Extreme unprofessional behaviour		Ever	Never	Missing
Inappropriate or unwanted touching		22 (4.2%)	506 (95.7%)	1 (0.2%)
Threats of violence/physical abuse		15 (2.8%)	513 (97.0 %)	1 (0.2%)
Physical assault		6 (1.1%)	523 (98.9%)	0 (0.0%)
Sexual assault		3 (0.6%)	525 (99.2%)	1 (0.2%)
Demands for sexual favours		2 (0.4%)	527 (99.6%)	0 (0.0%)

### Staff who most frequently reported experiencing unprofessional behaviour

**Fig. 1:**
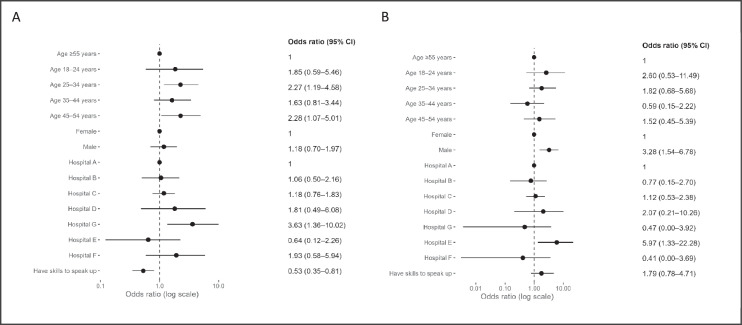
Allied health professionals’ experiences of incivility or bullying (A) and extreme unprofessional behaviour (B) by respondent characteristics and hospital.

Respondents aged 25–34 years and those 45–54 reported experiencing incivility or bullying more than those aged 55 and over (odds ratio (OR), 2.27; 95% CI, 1.19–4.58; OR, 2.28; 95% CI, 1.07–5.01) (Fig. 1). The odds for male and female respondents were similar for the experience of incivility or bullying. Males reported experiencing extreme unprofessional behaviour more than females (OR, 3.28; 95% CI, 1.54–6.78).

### Perceived skills in speaking up

Respondents who reported having speaking-up skills experienced frequent incivility or bullying less often than those who did not report having speaking-up skills (OR, 0.53; 95% CI, 0.35–0.81) (Fig. 1). Speaking-up skills were not associated with the experience of ‘extreme’ behaviours.

### Factors influencing comfort to speak up

**Fig. 2:**
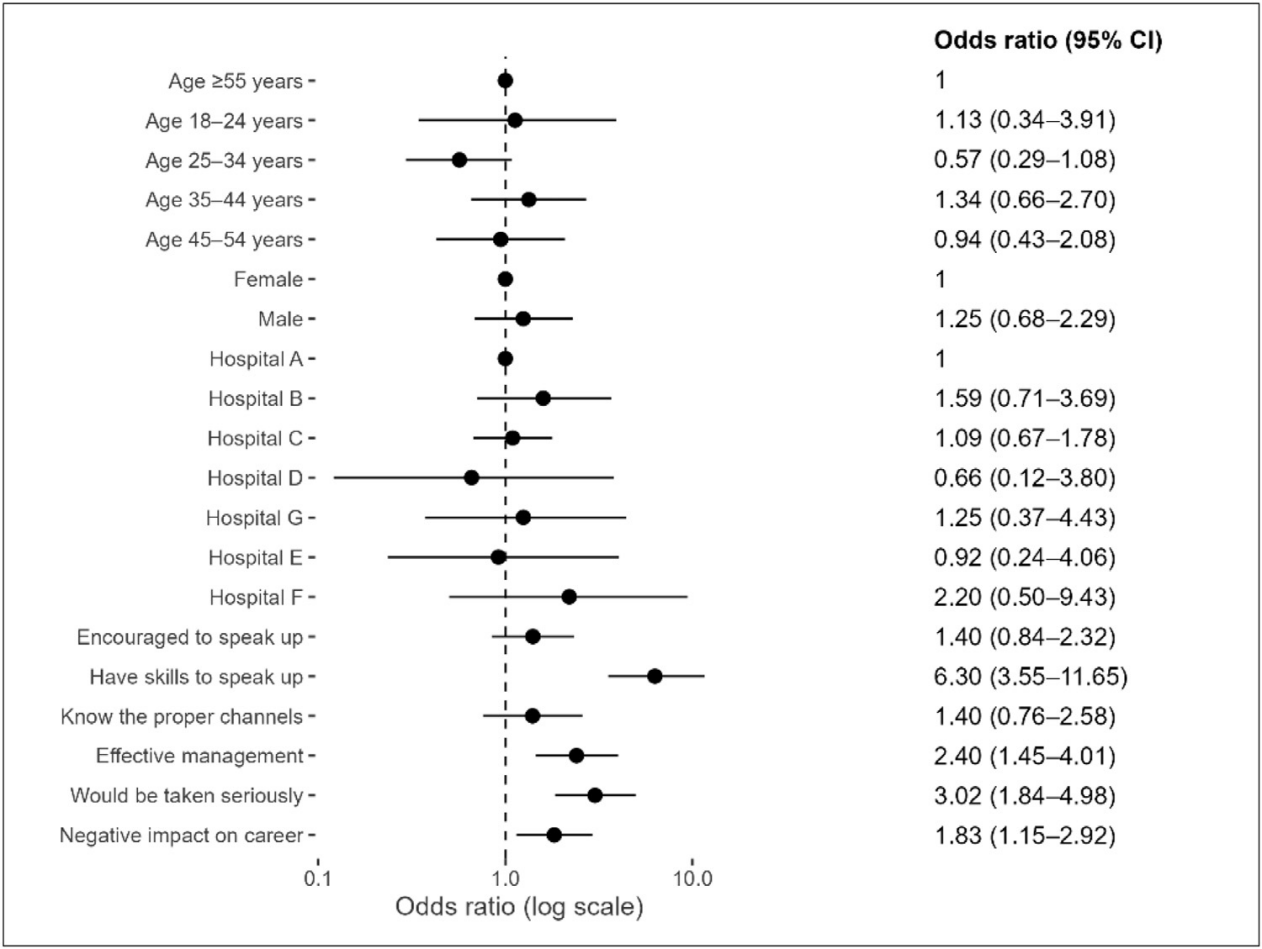
Allied health professionals’ comfort with speaking up and reporting unprofessional behaviour by respondent characteristics, hospital, and perceptions of speaking up.

There were no differences in the odds of reported comfort in speaking up by age or sex (Fig. 2). Several factors influenced respondent comfort in speaking up or reporting unprofessional behaviour. These factors included the perception that they had the skills to effectively speak up (OR, 6.30; 95% CI, 3.55–11.65), confidence that they would be believed and taken seriously if they reported unprofessional behaviour (OR, 3.02; 95% CI, 1.84–4.98), and the perception that unprofessional behaviour was effectively managed in the hospital (OR, 2.40; 95% CI, 1.45–4.01).

## Discussion

This study examined allied health professionals’ experience of unprofessional behaviour and their speaking-up skills. Key findings demonstrate that incivility or bullying were frequently experienced by these staff, but those who reported having speaking-up skills experienced frequent incivility or bullying less often than those who did not report having speaking-up skills.

Approximately 92% of allied health professionals experienced incivility or bullying at least once in the preceding 12 months and 29.7% experienced incivility or bullying weekly to multiple times daily. The prevalence of unprofessional behaviour experienced by these staff is similar to that experienced by other staff at the same hospitals.^2^ Results from other professional groups indicate that 86.6% of doctors and 92.8% of nurses experienced incivility or bullying in the preceding 12 months, and 36.1% of doctors and 41.4% of nurses experienced incivility or bullying on a weekly or daily basis.^2^ These findings are also similar to an Australian study of 370 consultant surgeons and trainees, with 83% experiencing bullying at least once in the preceding 12 months and 38% weekly or daily.^29^

Incivility or bullying compared to extreme unprofessional behaviour were especially common, with ‘opinions being ignored’ and ‘being spoken to rudely’ frequently experienced by allied health professionals. Previous study findings are mixed as to differences in experiences of unprofessional behaviour across age groups.^30^, ^31^, ^32^, ^33^ We found that respondents aged 25–34 years reported experiencing incivility or bullying more than respondents aged 55 and over. Higher hierarchical status and longer healthcare service experience may reduce the likelihood of experiencing unprofessional behaviour. Although there were no differences between men and women for reported experiences of incivility or bullying, men reported experiencing extreme unprofessional behaviour more than women; however, the total number of extreme unprofessional behaviours reported in our study was small. Previous studies examining gender differences are mixed and may be dependent upon the combinations of behaviours grouped in this category.^31^^,^^34^, ^35^, ^36^

Unprofessional behaviour experienced by doctors and nurses has been shown to impair clinical performance and negatively impact patient outcomes.^13^^,^^37^ Allied health professionals work as part of multidisciplinary teams with doctors and nurses to coordinate patient care. It is likely, then, that the experiences of allied health professionals may also undermine patient safety. Previous research has focused on the importance of communication between doctors and nurses for clinical performance and quality of care.^38^ However, effective collaboration between these staff and allied health professionals is also important for the welfare of patients. Our findings demonstrate that allied health professionals frequently experienced ‘opinions being ignored’, suggesting their lower status on the hierarchy within clinical teams may lead to experiences of unprofessional behaviour. Allied health professionals may consequently feel unable to speak up to address unprofessional behaviour.

Speaking up about unprofessional behaviour is less normalised in health systems compared with speaking up about traditional patient safety concerns. A culture of speaking up in the workplace is influenced by a range of organisational and interpersonal factors, including hierarchy, norms, leadership and workplace histories.^19^^,^^39^ Previous studies have found that hospital staff perceive speaking up about unprofessional behaviour as unlikely to lead to change, more confrontational, and comprising greater risks compared with raising concerns about patient safety.^40^ Male resident doctors (ie medical school graduates with a Doctor of Medicine degree undertaking postgraduate training) have been found to be more likely to speak up about safety issues related to professionalism than female residents.^41^ In our study, we did not find differences between male and female allied health professionals for reported comfort in speaking up. Staff aged 25–34 years were less likely to feel comfortable speaking up compared with those aged 55 years and over. This may be due to older staff having more workplace experience and more advanced communication skills. They may also have higher hierarchical status and consequently could be less likely to experience unprofessional behaviour. Younger allied health professionals may need additional support and skills training to develop their speaking-up skills.

Speaking-up programmes that aim to address unprofessional behaviour have been implemented in hospitals in several countries.^18^ The effectiveness of these programmes to improve staff speaking-up skills and whether these skills reduce the frequency and impact of unprofessional behaviour is unclear. Our findings indicate that allied health respondents with self-reported speaking-up skills were 47% less likely to report experiencing frequent incivility or bullying compared with respondents without these skills. This finding suggests that speaking-up skills may assist staff to address unprofessional behaviour supporting the investment of health systems in speaking-up programmes. These programmes need to be tailored to hospital staff who are at high risk of experiencing unprofessional behaviour if they are to be successful. Effectiveness of these interventions is also dependent on organisations simultaneously addressing the underlying drivers of unprofessional behaviour.^11^

Our study is one of few to examine unprofessional behaviour experienced by allied health professionals. Staff from seven hospitals across three Australian states completed our survey. A strength of our study is we performed a rigorous modelling approach to investigate the outcomes of interest. A limitation is the data were collected in 2017–2018. However, the reported prevalence of unprofessional behaviour experienced by allied health professionals in our sample is similar to those reported in previous as well as more recent studies of hospital staff, suggesting that unprofessional behaviour continues to be a persisting issue.^4^^,^^42^^,^^43^ Another limitation is that we did not ask respondents about characteristics such as ethnicity, country of origin, religion, sexual orientation, relationship status, pregnancy or disability. We also did not include items about the professional group of perpetrators. Future research on potential differences in the experiences of minoritised groups can provide further direction on how interventions can be developed to better support these staff groups. Responder bias was possible in our study, as staff who had experienced unprofessional behaviour may have been more likely to complete the survey. The study was a cross-sectional survey and thus findings can only demonstrate associations.

Allied health professionals are at a similar risk of experiencing unprofessional behaviour from co-workers as other hospital staff such as doctors and nurses. The frequent experience of unprofessional behaviour by allied health staff may negatively impact their performance in clinical teams and potentially jeopardise the safety of patients. Our findings suggest that programmes that support staff to speak up as a component within organisational safety culture programmes may be beneficial to assist in addressing unprofessional behaviour.^42^ Further research is required to evaluate the effectiveness and sustainability of such programmes for all staff if culture change is to occur in hospitals.

## CRediT authorship contribution statement

**Ryan D. McMullan:** Writing – original draft, Project administration, Methodology, Investigation, Data curation, Conceptualization. **Tim Badgery-Parker:** Writing – review & editing, Formal analysis, Data curation. **Ling Li:** Writing – review & editing, Formal analysis. **Rachel Urwin:** Writing – review & editing. **Johanna I. Westbrook:** Writing – review & editing, Resources, Methodology, Conceptualization.

## Appendix Supplementary materials

Image, application 1
