# Supplementary material for: Allied health professionals’ experiences of co-worker unprofessional behaviour and their reported speaking-up skills: A secondary analysis of a cross-sectional survey
Source: Future Healthc J. 2025 Jun 6;12(3):100262. doi: 10.1016/j.fhj.2025.100262 (PMC12206027; doi:10.1016/j.fhj.2025.100262)
Supplement: Supplementary file 1 [file mmc1.docx]

1. **Longitudinal Investigation Of Negative behaviour (LION)**

**Survey questions relevant to this report**

**Demographics**

Age (*18-24; 25-34; 35-44; 45-54; 55-64; 65+ years; Prefer not to answer)*

Gender (*Female, Male, Other, Prefer not to answer)*

Role

*Medical (e.g. Staff specialist/VMO; Registrar; Career/Hospital Medical Officer; Resident; Intern)*

*Nursing (e.g. Nursing Unit Manager; Clinical Nurse Consultant; Nurse Educator; Registered Nurse or Midwife; Enrolled Nurse; Graduate Nurse or Midwife)*

*Allied Health & Clinical Services (e.g. Pharmacy, Physiotherapy; Occupational Therapy; Dietitian; Psychologist; Medical imaging; Pathology collector; Social worker; Technologist)*

*Non-Clinical Services (e.g. Scientist/laboratory staff; Personal care assistant; Orderly; Food services; Engineering services; Environmental services; Security; Tradesperson)*

*Management & Administrative (e.g. Manager; Finance officer; Human Resources; Medical Records; IT; Ward Clerk; Patient Services Clerk)*

Length of employment in current hospital *(Less than 1 year; 1-2 years; 3-5 years; 6-10 years; 11-20 years; Over 20 years)*

Length of employment in healthcare sector *(Less than 1 year; 1-2 years; 3-5 years; 6-10 years; 11-20 years; Over 20 years)*

**Unprofessional behaviour questions**

In the past 12 months, how often have you experienced or seen the following staff behaviours in this hospital:

Response options:

*Never*

*1-2 times/year*

*Every few months*

*Around monthly*

*Weekly*

*Daily*

*Multiple times daily*

*Prefer not to answer*

1. Being spoken to rudely
2. Someone withholding information which affects work performance
3. Opinions being ignored
4. Being shouted at or being the target of anger
5. Being told sexually explicit or offensive jokes/ comments at work
6. Physically intimidating behaviours (e.g. finger-pointing, invasion of personal space, blocking)
7. Hints or signals from others to quit your job
8. Repeated reminders of errors or mistakes
9. Excessive monitoring of work
10. Unwelcome practical jokes
11. Being given unreasonable workload/deadlines
12. Graphic comments/ questions/ insinuations about appearance, sexual or private life
13. Being the subject of excessive teasing/sarcasm
14. Threats of violence/physical abuse
15. Being ignored or excluded
16. Inappropriate or unwanted touching
17. Unwelcome sexual flirtations/persistent requests for dates
18. Being humiliated or ridiculed
19. Demands for sexual favours
20. Having unjustified allegations made
21. Having key areas of responsibility removed or replaced with meaningless or unpleasant tasks
22. Being shown sexually suggestive photos, videos, emails or texts
23. Sexual assault
24. Physical assault (e.g. hitting, shoving, punching)
25. Negative comments or offensive jokes about gender, ethnicity, sexual orientation, religion, disability, pregnancy, parenting responsibilities, age

- If yes, comments/jokes were based on (choose all that apply):

Gender

Ethnicity

Sexual orientation

Religion

Disability

Pregnancy

Parenting/carer responsibilities

Age

1. Treated unfairly based on gender, ethnicity, sexual orientation, religion, disability, pregnancy, parenting responsibilities, age

- If yes, comments/jokes were based on (choose all that apply):

Gender

Ethnicity

Sexual orientation

Religion

Disability

Pregnancy

Parenting/carer responsibilities

Age

**Speaking up questions**

Thinking about unprofessional staff behaviours in this hospital, how much do you agree or disagree with the following statements:

Response options:

*Strongly disagree*

*Disagree*

*Neither disagree nor agree*

*Agree*

*Strongly agree*

1. Speaking up or reporting unprofessional behaviour is important for patient safety
2. I am encouraged by my colleagues to speak up about unprofessional behaviour
3. I have the skills to effectively speak up if I experience unprofessional behaviour
4. I have the skills to effectively speak up if others experience unprofessional behaviour
5. I know the proper channels to raise concerns about unprofessional behaviour
6. Unprofessional behaviour is effectively managed in this hospital
7. I feel comfortable speaking up or reporting unprofessional behaviour
8. It takes too much time and effort to report unprofessional behaviour
9. I am confident I would receive support from my supervisor if I reported unprofessional behaviour
10. Speaking up or reporting unprofessional behaviour is likely to have a negative impact on my career
11. I am confident I would be believed and taken seriously if I reported unprofessional behaviour
